# Norfolk and Norwich Hospital

**Published:** 1919-05-24

**Authors:** Frank Inch

**Affiliations:** *Secretary.* Norfolk and Norwich Hospital, Norwich.


Yours faithfully,

Gladys M. E. Leigh.

### Norfolk and Norwich Hospital.

Sir,—With reference to the paragraph headed "Home for Hospital Servants," appearing in the issue of The Hospital dated May 10, I have to point out that the statement, "it will consist of 24 rooms," is incorrect. As a matter of fact, there will be 80 rooms in the home, one for each maid, with one or two spare rooms for pupil housekeepers.

Further, the appeal to be issued is for the sum of £30,000, and the intention is to pull down the remaining wing of the old hospital and rebuild an up-to-date orthopædic department and out-patient department (both of which improvements are urgently required), in addition to the Maids' Home.—Yours faithfully,

Frank Inch, *Secretary*.
Norfolk and Norwich Hospital, Norwich.

[The number "24" is what Mr. Horsfall is lately reported to have said. Probably he was referring to the number of maids' bedrooms only. The complete scheme is deservedly a comprehensive one.—Ed. The Hospital.]

